# Construction of China cardiovascular health index

**DOI:** 10.1186/s12889-018-5647-7

**Published:** 2018-07-31

**Authors:** Yingying Jiang, Fan Mao, Yichong Li, Jing Liu, Yan Zhang, Yong Jiang, Dong Zhao, Weiwei Chen, Stephen Nicholas, Yong Huo, Junbo Ge, Linhong Wang, Maigeng Zhou

**Affiliations:** 1 National Center for Chronic and Non-communicable Disease Control and Prevention, Chinese Center for Disease Control and Prevention, 27 Nanwei Road, Xicheng District, Beijing, 100050 China; 2 Peking University Clinical Research Institute, Beijing, China; 3 Beijing Institute of Heart Lung and Blood Vessel Diseases, Beijing Anzhen Hospital, Capital Medical University, Beijing, China; 4 Peking University First Hospital, Beijing, China; 5 Beijing Tiantan Hospital, Capital Medical University, Beijing, China; 6 National Center for Cardiovascular Disease, Beijing, China; 7 School of Management and School of Economics, Tianjin Normal University, West Bin Shui Avenue, Tianjin, 300074 People’s Republic of China; 8 Guangdong Research Institute for International Strategies, Guangdong University of Foreign Studies, 2 Baiyun North Avenue, Baiyun, Guangzhou, Guangdong 510420 People’s Republic of China; 9 School of International Business, Beijing Foreign Studies University, 19 North Xisanhuan Avenue Haidian, Beijing, 100089 People’s Republic of China; 10 University of Newcastle, Newcastle, NSW 2308 Australia; 11 Zhongshan Hospital, Fudan University, Shanghai, China

**Keywords:** China cardiovascular health index (CHI), Index system, Delphi method, Analytical hierarchy process (AHP) model, Weight, Monitor

## Abstract

**Background:**

Cardiovascular disease (CVD) is not only the primary cause of death in developed western countries, but also its disease burden is increasing in China. The purpose of constructing population cardiovascular health index is to monitor, compare and evaluate disease burden, influencing factors and prevention and control levels of Chinese population cardiovascular disease in order to provide evidence to improve population cardiovascular health.

**Methods:**

This study collected multi-source data and constructed China Cardiovascular Health Index (CHI) using literature review, questionnaire surveys, Delphi method and Analytical Hierarchy Process (AHP) model.

**Results:**

China CHI system included 52 indices of 5 dimensions, which were prevalence status of CVD, exposure of risk factors, prevention and control of risk factors, treatment situation and public health policy and service ability. The weights of 5 dimensions from high to low were successively prevention and control of risk factors 0.3656, prevalence status of CVD 0.2070, treatment situation 0.1812, public health policy and service ability 0.1458, and exposure of risk factors 0.1004.

**Conclusion:**

China CHI is a comprehensive evaluation system raised to effectively control the prevalence of CVD. In the future, we should strengthen and improve CVD monitoring and big data usage, to ensure these indices to reflect the practical situations and to become utility of controlling CVD.

## Background

With 17.9 million deaths, the 2015 Global Burden of Disease (GBD) study reported that cardiovascular disease (CVD) was the primary cause of deaths worldwide. Among them, 15.2 million people died of ischemic heart disease (IHD) and stroke, accounting for 85.1% of the total CVD deaths [1]. CVD is not only the primary cause of death in developed western countries, but also its disease burden is increasing in China. With lifestyle changes linked to economic development, population ageing and accelerating urbanization, CVD risks have risen significantly in China over the past 30 years. For example, the mortality of CVD was 271.8/100000 in 2015. The prevalence of cerebral vascular disease increased from 9.8% in 1993 to 12.3% in 2013. The prevalence of hypertension in Chinese residents ages 18 years and above was 25.2% which was on the rise compared with that of 2002 [2, 3]. The proportion of China’s IHD disability adjusted of life years (DALYs) increased from 3.59% in 1995 to 6.24% in 2015, and the proportion of stroke increased from 8.15 to 10.11%. In 2013, 3.72 million Chinese people died of CVD, about 46% more than the 2.56 million CVD deaths in 1990. During the same period, the IHD death toll rose 91% from 0.745 million to 1.394 million [4]. China’s most recent CVD report estimated that 290 million people suffered from CVD [5], with fatality and mortality rates higher in rural areas than those in urban areas since 2009. The report also showed that inpatient CVD expenses had increased faster than the growth in GDP since 2004 [5]. Today, CVD is the single greatest risk factor facing Chinese people’s health.

The prevention and control of CVD is a gradual process, requiring either individual interventions, especially through education, that change lifestyles or expensive medical interventions. Long-term and sustained prevention measures need action at both the global and national levels, with the initial step the effective evaluation of the prevalence of CVD, CVD risks and the effectiveness of CVD prevention and control. To implement this initial step, monitoring systems and relevant indices must be established to measure the extent and change in CVD. In the Global Action Plan for the Prevention and Control of NCDs 2013–2020, the World Health Organization (WHO) indicated that with effective monitoring early deaths caused by CVD, tumors, diabetes and chronic obstructive pulmonary disease (COPD) could be reduced by 25% [6]. The American Health Association’s (AHA) 2020 goals to improve Americans’ CVD health status, called for preventative measures to reduce CVD stroke deaths by 20%. Many Chinese health policies, such as Healthy China 2030 and China’s Medium and Long Term Plan of Preventing and Controlling Chronic Diseases, also called for the reduction of critical chronic diseases deaths [7, 8]. For example, China’s Medium and Long Term Plan of Preventing and Controlling Chronic Diseases set the goal to reduce CVD mortality rates by 10% by 2020 and 15% by 2025 and Healthy China 2030 promoted a 10% reduction in probability of premature deaths by 2020 and 30% by 2030, compared with 2015 rates.

To realize China’s national goals of CVD prevention and control, effective evaluation and monitoring measures of China’s cardiovascular health status are required. While the proportion of percutaneous coronary intervention (PCI) among patients with ST segment Elevation Myocardial Infarction (STEMI) can reflect CVD treatment, the index does not measure population cardiovascular health status. Although the probability of premature deaths is a commonly used index proxing the prevalence of CVD, it is both poorly understood by the public and ineffective in predicting individual-level intervention and education. What is needed is a simple and direct evaluation index system that reflects the Chinese people’s cardiovascular health status; monitors, compares and evaluates the prevalence, severity and control level of CVD; and measures the effectiveness of prevention and treatment of people with CVD. An effective index system should provide a significant reference point for issuing and implementing health policies, so as to gradually monitor the improvement in the Chinese people’s health expectancy and to effectively control the CVD burden.

Importantly, an innovative Chinese CVD index should facilitate policy makers understanding of China’s current CVD situation; improve the general public awareness of CVD; and help health professionals find breakthrough points in improving CVD rates. We propose a cardiovascular health index (CHI) for China that collects all relevant data about cardiovascular health to objectively, scientifically and impartially reflect the cardiovascular health status of Chinese population. Specifically, this paper describes the establishment of the CHI, specifying the methods for constructing various sub-indices, and also proposes a future research plan using the CHI.

## Methods

**Fig. 1:**
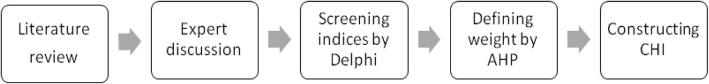
Process of constructing of CHI

In order to evaluate the Chinese population’s cardiovascular health status, a comprehensive CHI should reflect the current prevalence of CVD and its risks; provide measures for preventing and treating CVD; and emphasize public health policies and basic population and community interventions. To construct the CHI, we undertook a literature review, sought expert consultations and utilized the Delphi Method and the analytical hierarchy process (AHP) method. Figure 1 diagrammatically displays the steps in the CHI construction from literature review, expert discussion, index screening and weight defining to the final CHI.

### Define CHI dimensions

In our review of the prevention and treatment of CVD literature, there was no paper providing a comprehensive index of cardiovascular health, which suggests that constructing a China-based CHI is innovative. While we discovered no comprehensive CVD indices as a reference point, pointers were provided by documents issued by American Heart Association (AHA), the NCD Global Monitoring Framework published by the WHO, which included indices that evaluated the CVD burden, CVD monitoring and prevention, the control of CVD risks and CVD treatments. When constructing the CHI, we took these indices as frames of reference, following the idea of “prevention first, and prevention and treatment integrated” from the AHA, ACC and WHO [9–11]. We also referenced Chinese health policies, such as Healthy China 2030, which proposed that CHI development should include an evaluation of CVD prevalence and the proposal and implementation of targeted CVD measures. Finally we held expert discussions to define CHI dimensions.

### Defined the CVD index system using Delphi method

**Fig. 2:**
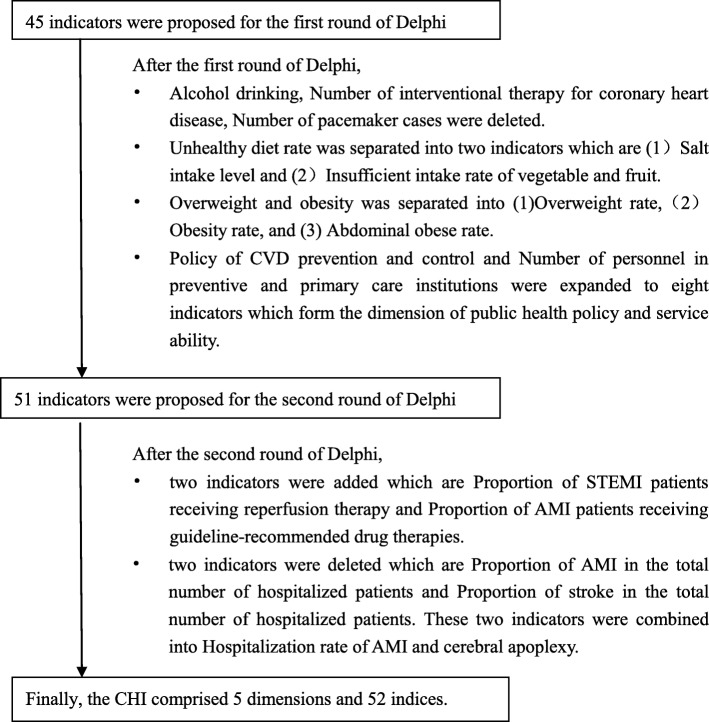
Flowchart of Delphi process

Through the literature review, we established an index base and derived a pilot survey, which was used to develop a questionnaire for expert consultation. Next, we defined the indices through two-round Delphi method [12]. First, professionals specializing in chronic disease prevention and control and clinical experts working on treating CVD participated in group consultations. Second, experts marked the significance of each index (from 1 equal importance to 9 highest degree of importance), and listed the reasons and evidence in the memo field. If its median score ≥ 8 and inter-quartile rage ≤2, the index was accepted. Finally, the second-round Delphi method was conducted, where the consultation group calculated the median and inter-quartile range of each index, excluding those indexes that did not meet the inclusion criteria, and adding new indices based on expert suggestions. SAS software was used to do statistical analysis. The flowchart of Delphi process was shown as Fig. 2.

### Defining the CHI weights with AHP

**Fig. 3:**
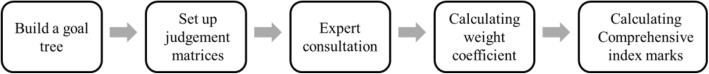
Procedure of AHP

We adopted the analytical hierarchy process (AHP) [13] to define the dimensions and weight of each cardiovascular health index. First, we stratified the indices and set up a goal tree, where the indices within each dimension were stratified to form a hierarchical structure and a CHI weight evaluation goal tree was constructed. Next, we set up judgment matrices. According to the hierarchical structure of the goal tree, we built up pair-wise comparisons of the judgment matrices to define the relative significance of each individual index, and designed a CHI weight questionnaire for expert consultation. For the expert panel, we selected 11 senior experts specializing in prevention and treatment of CVD, including expertise in epidemiology and statistics, prevention and control of chronic disease, clinical cardiovascular medicine, epidemiology of pulmonary vascular disease, epidemiology of cerebrovascular disease, policies, and public health administration. The expert panel enumerated the judgment matrices (see Table 1) in the questionnaire according to the CHI weights, and marked the goal tree based on pair-wise comparisons, using the evaluation criteria shown in Table 2. For indices at the same level, a certain weight was given individually according to its value on the indices at the upper level. YAAHP software was applied to the expert scoring process. The consistency of the expert judgment matrix was calculated to test the logic of the judgment. Generally, when consistency index CI (or random consistency ratio CR) was less than 0.10, the judgment matrix was strongly consistent, and the weight coefficient met logic consistency. If the consistency was not satisfied, the expert were asked to repeat the scoring process until the consistency of each matrix was satisfied, and the expert were able to submit the result of the matrix scoring. Based on the experts’ marks, the study calculated the initial and normalized weight coefficients within each level and the combination weight coefficient of each index. According to the principle that each expert’s mark was equally weighted, we averaged the experts’ marks, and calculated the final weight of each level and that of each index. Procedure of AHP was shown in Fig. 3.

**Table 1:** Examples of judgment matrices marking

	Probability of premature deaths of total vascular disease	Probability of premature deaths of cerebral apoplexy	Probability of premature deaths of coronary heart disease
Probability of premature deaths of total vascular disease	1(a_11_)	3(a_12_)	5(a_13_)
Probability of premature deaths of cerebral apoplexy	1/3(a_21_)	1(a_22_)	3(a_23_)
Probability of premature deaths of coronary heart disease	1/5(a_31_)	1/3(a_32_)	1(a_33_)

The above scores are not real marks used in AHP for evaluating CHI weights

**Table 2:** Marking criteria of each level of the goal tree

Marks	Relative importance	Interpretation
1	Equal importance	The two contribute the same to the goal.
3	Slight importance	One is slightly more important than the other based on experience.
5	Basic importance	One is more important than the other based on experience.
7	Strong importance	One is strongly more important than the other, and it is proved in practice.
9	Absolute importance	One is absolutely more important than the other.
(2,4,6,8)	The median of two adjacent levels	Adopted for compromising.

### Calculation of CHI marks

Based on indices selected using the Delphi method and the index weights defined by AHP, the CHI was constructed to calculate the cardiovascular health indices in each region of China. Region-based CHIs allowed comparisons across regions diverse in population size, economic development, urban-rural mix and different health regimes, which assisted public health policy-making. The full mark of each index was 100. The higher the score for any region, the better the cardiovascular health status.

Since individual indices differed greatly in dimensions, magnitudes and contents, we undertook index co-directional transformation, standardization and percentage transformation of the aggregate CHI. First, we retained all indices towards the same direction. In the multi index comprehensive evaluation, some indicators are called the positive index that means the greater the index value is, the better the evaluation is; some indicators are called reverse index that means the smaller the index value is, the better the evaluation is. In the comprehensive evaluation, it is necessary to first turn the index into the same direction. It is also called the positive of the index. This decision rule realized that the higher the index value, the better the sanitary conditions, services or environment related to cardiovascular health. As for the indices like probability of premature deaths and prevalence rate of risk factors, we negativity transformed the index by multipling by − 1. For each index, the higher their values, the better the health conditions. Secondly, we standardized the indices. A preliminary analysis showed that most of the indicators were subject to normal distribution. Therefore, the standard normal transformation was used to remove the dimension of each index, so that all transformed indexes obey the standard normal distribution with a mean value of 0 and a standard deviation of 1. The transformation formula was:

$$ {z}_i=\frac{X_i-{\mu}_i}{\sigma_i} $$

*z*_*i*_ refers to the mark of ith index after standard normal transformation, *X*_*i*_ refers to the initial or co-directional value of the ith index, *μ*_*i*_ refers to the mean value of ith index of each China province, *σ*_*i*_ refers to the standard deviation (SD) of ith index of each province.

To ensure that the final value fell between 0 and 100, it was necessary to undertake percentage transformations. This was done by calculating the area under the standard normal distribution curve on the left side of value z. As for any standard normalized index *z*_*i*_, its score was S_*i*_:

$$ {\mathrm{S}}_i=100\bullet {\int}_{-\infty}^{z_i}\frac{1}{\sqrt{2\pi }}{e}^{-\frac{x^2}{2}} dx $$

Finally, combining each index’s standard normalized mark and weight, we calculated the marks of different dimensions in each province and final CHI marks. The calculation method was:

$$ \mathrm{CHI}=\sum \limits_{i=1}^n{S}_i\cdot {w}_i $$

*n* refers to the number of indices of a certain dimension or the number of all indices, *S*_*i*_ refers to the marks of standard normalization, and *w*_*i*_ refers to index weight.

**Table:** 

Taking the smoking rate as an example of the calculation: The smoking rate (%) of Province A is 24.6, the average value of 31 provinces is 26.86 and the standard deviation is 3.53. The weight of this indicator is 0.0136. The score of smoking rate of Province A is calculated as follows: • co-directional transformation of the smoking rate which means the value (24.6)need to be multiplied a − 1. • Z_smoking_ = [− 24.6-(− 26.86)]/3.53 = 0.64062919 • S _smoking_ = 73.9 • S_smoking weighted_ = 73.9*0.0136 = 1.005200734 The calculation of the remaining indicators has the similar procedure as smoking rate. The total score of CHI is obtained by accumulating the scores of all indicators.	

## Results

### CHI dimensions and indices

Considering that the CHI is a comprehensive index system, it should reflect integrity and include concrete and complete indices. The comprehensive exponential model is usually complex and uncertain, so it was necessary to include different kinds of factors influencing that index and to reflect instantaneity, stability and continuity of the indices. Therefore, we established an index system comprising 5 dimensions: prevalence of CVD, exposure of risk factors, prevention and control of risk factors, treatment of CVD and public health policy and service ability. These dimensions allowed different CVD researchers to focus on specific elements. Through literature reviews, we selected in total of 41 relevant indices and built up an index base.

### Screening of indices

During the first round of Delphi method, we handed out 155 questionnaires (34 in the field of public health and 121 for clinical experts), and received back 104 questionnaires (24 in the field of public health and 80 for clinical experts). The response rate was 67%, with respondents’ average age 53.3 years and 92.9% holding a senior technical titles. During the second round of Delphi method, we handed out 133 questionnaires (34 in the field of public health and 99 for clinical experts), and received back 73 questionnaires (22 in the field of public health and 51 for clinical experts). The response rate was 55%, with experts’ average age of 54.9 years and all of them held a senior technical title.

According to the index screening principle, the CHI comprised 5 dimensions and 52 indices after two rounds of the Delphi method (See Table 3). The data sources of these indicators included Global Burden of Disease China (2015), Chronic Disease Risk Factor Surveillance in China 2013, China Health and Family Planning Yearbook 2016, Chinese National Stroke Registry, Traffic Management Bureau of the Public Security Ministry, Reporting System of China Chest Pain Center Certification, Improving Care for Cardiovascular Disease in China, National Mortality Surveillance System in China and China Drug Supply Information Platform.

**Table 3:** Indices and weights of CHI

Stair index (dimension)	Secondary index	Number	Index	Weight
A. Prevalence of CVD	A1 Probability of premature deaths	A01	Probability of premature deaths from the total cardiovascular disease	0.0750
		A02	Probability of premature deaths from cerebral apoplexy	0.0315
		A03	Probability of premature deaths from coronary heart disease	0.0309
	A2 Morbidity rate	A04	Morbidity rate of myocardial infarction	0.0348
		A05	Morbidity rate of cerebral apoplexy	0.0348
B. Exposure of risk factors	B1 Behavior	B01	Smoking rate	0.0136
		B02	Physical inactivity rate	0.0083
		B03	Salt intake level (g/d)	0.0071
		B04	Insufficient intake rate of vegetable and fruit	0.0061
	B2 Metabolic indices	B05	Overweight rate	0.0009
		B06	Obesity rate	0.0024
		B07	Abdominal obese rate	0.0034
		B08	Morbidity rate of hypertension	0.0216
		B09	Morbidity rate of diabetes	0.0123
		B10	Morbidity rate of hyperlipoidemia	0.0093
	B3 Concentration of PM2.5	B11	Concentration of PM2.5	0.0152
C.Prevention and control of risk factors	C1 Hypertension	C01	Blood pressure detection rate	0.0270
		C02	Awareness rate of hypertension	0.0327
		C03	Treatment rate of hypertension	0.0506
		C04	Control rate of hypertension	0.0684
	C2 Diabetes	C05	Blood glucose detection rate	0.0131
		C06	Awareness rate of diabetes	0.0133
		C07	Treatment rate of diabetes	0.0175
		C08	Control rate of diabetes	0.0255
	C3 Hyperlipoidemia	C09	Blood lipid detection rate	0.0121
		C10	Awareness rate of hyperlipoidemia	0.0104
		C11	Treatment rate of hyperlipoidemia	0.0148
		C12	Control rate of hyperlipoidemia	0.0204
	C4 Successful smoking cessation rate	C13	Successful smoking cessation rate	0.0596
D. Treatment of CVD	D1 Treatment ability	D01	Number of doctors at cardiovascular department and neurology department	0.0101
		D02	Number of catheter room	0.0053
		D03	Number of ambulance	0.0067
		D04	Number of beds at cardiovascular department and neurology department	0.0079
		D05	Number of chest pain center	0.0058
		D06	Number of stroke center	0.0056
	D2 Treatment procedure	D07	Proportion of primary PCI on total PCI procedures among STEMI patients	0.0054
		D08	Proportion of thrombolysis among ischemic stroke patients	0.0047
		D09	Proportion of STEMI patients receiving reperfusion therapy	0.0060
		D10	Proportion of AMI patients receiving guideline-recommended drug therapies	0.0057
		D11	Hospitalization rate of AMI and cerebral apoplexy	0.0194
	D3 Treatment outcome	D12	In-hospital mortality of AMI patients	0.0203
		D13	In-hospital mortality of cerebral apoplexy patients	0.0203
		D14	Out-of-hospital death of AMI and cerebral apoplexy to total death of cardiovascular and cerebrovascular diseases	0.0227
		D15	Death of AMI and cerebral apoplexy to total death	0.0351
E. Public health policy and service ability	E1 Policy	E01	Intervention policy of risk factors	0.0245
		E02	Security policy of chronic diseases	0.0274
	E2 Health expenditure	E03	Proportion of government input on CVD	0.0142
		E04	Use of medicine interfering risks	0.0160
	E3 Residents’ health literacy level	E05	Residents’ health literacy level	0.0291
	E4 System construction of prevention and control	E06	Availability of affordable basic technologies and essential medicines	0.0160
		E07	Number of CDC professionals	0.0056
		E08	Number of General practitioners	0.0130

### Index weight

According to the results of AHP, the weights of 5 dimensions from high to low were prevention and control of risk factors 0.3656, prevalence of CVD 0.2070, treatment of CVD 0.1812, public health policy and service ability 0.1458 and exposure of risk factors 0.1004. Within the prevalence of CVD, the weight of probability of premature deaths (0.1374) was higher than that of morbidity rate (0.0696). Within exposure of risk factors, the weight coefficients of secondary indices from high to low were metabolic indices (0.0501), behavior (0.0351) and concentration of PM2.5 (0.0152). Within prevention and control of risk factors, the weight coefficients of secondary indices from high to low were hypertension (0.1787), diabetes (0.0695), successful smoking cessation rate (0.0596) and hyperlipoidemia (0.0577). Within the prevalence of CVD, the weight coefficients of secondary indices from high to low were treatment outcome (0.0985), treatment ability (0.0414) and treatment procedure (0.0413). Within public health policy and service ability, the weight coefficients of secondary indices from high to low were policy (0.0520), system construction of prevention and control (0.0346), health expenditure (0.0302) and residents’ health literacy level (0.0291). The weights of each index are listed in Table 3.

## Discussion

This study is carried out by multi center cooperation. The team researchers come from the National Center for Noncommunicable Disease Control and Prevention, heart disease specialist hospitals, stroke specialist hospitals, and health administration departments. All the data required to calculate the CHI are from high-quality research such as Research on Provincial Burden of Disease and Improving Care for Cardiovascular Disease in China (CCC Research), and official released Chinese data (such as the health statistics yearbook of the National Health Commission, the national surveillance on chronic disease and risk factors for adult in China, the Traffic Management Bureau). These data are the most representative data so far which are supposed to ensure the consistency and stability of the research. CHI adopted the comprehensive index method, applying the exponentiation of indices and comprehensive comparison among indices with different characteristics, categories and measurement units. We employed the commonly used Delphi methods for constructing comprehensive indices [14–17], and undertook a literature review and Delphi method that are most commonly used in evaluating and monitoring the prevention and control system of chronic disease [10]. Based on literature review, we combined qualitative and quantitative analysis, summarized experts’ back-to-back individual evaluation results and face-to-face group evaluation opinions by using Delphi method and AHP, and finally established the CHI system and gained the weight of each index.

In the considering the advantages of the CHI, 3 core concepts were taking into account. First, the power of comprehensive evaluation. The CHI dimensions and indices reflect the need to adapt comprehensive methods to evaluate population cardiovascular health in an ideal state. The CHI includes indices for evaluating clinical doctors’ job quality, indices at population level to reflect the effect of healthy lifestyle on improving cardiovascular health, health behavior indices (e.g. smoking rate, overweight and obesity rate, insufficient physical activity rate, insufficient intake rate of vegetable and fruit and intake level of salt), influencing factors of cardiovascular health (e.g. blood lipid, blood pressure and blood sugar), indices about clinical treatment, and intervention indices based on public health, community and population. The CHI is a concept with multi-dimension and multi-indexes. For China, the CHI is comprised of 30% weights reflecting the prevalence of CVD and 70% weights of three dimensions reflecting measures against CVD.

Second, the concepts of prevention and the importance of risk factors prevention. As for the stair indices, the weight of prevention and control of risk factors was the highest accounting for 36%. Within this dimension, the weight of hypertension controlling rate was 0.0684, taking up the highest proportion; followed by successful smoking cessation rate (weight 0.0596) and treatment rate of hypertension (weight 0.0506). Weights of control rate of hypertension, successful smoking cessation rate and treatment rate of hypertension listed respectively 2–4 among the 52 CHI indices, where the first weight was probability of premature deaths of total vascular diseases with a weight of 0.075. Risks intervention and high risk population intervention were the most important area of population CVD prevention and treatment [18]. Successful experience in risks intervention affirms that risks intervention was effective in reducing morbidity and mortality rates of CVD, and this effect applies to the whole population and individual’s full life circle. For example, half years after Helena Mont Smoke-free Regulation was issued, hospital ACI mortality decreased by 50% year-on-year [19]. There are further examples, such as the studies about salt reduction on reducing blood pressure levels [20–22], studies about improving physical activities by building supporting environment, projects about building environment in Finland and China Shougang Group CVD prevention and control project [23–25]. As a developing country, China is relatively deficient in health resources, while prevention and control of risk factors has been proved to be the most cost-effective intervention and the most urgent action in the field of disease control [25]. The results show that the weight of prevention and control of risk factors was the highest, indicating that prevention and control of risk factors is crucial to preventing and controlling CVD among Chinese population. Moreover, in the dimension of public health policy and service ability, the weight of policy was the highest (35%). Considering that health issues are closely related with social and economic factors, Health in All Policies (HiAP) [26] is regarded as an important strategy to solve issues related to health. Our results show that the weights of policy indices were comparatively higher, which agree with that strategy.

Last, the importance of monitoring. WHO study showed that when dealing with prevalence of chronic disease, low and middle income countries performed worse in monitoring and researching disease and risk intervention [10]. This also applied to China. There is a pandemic of CVD in China, led the China government to issue political statement on the need to counter the pandemic [7, 8]. A set of targets, including reducing deaths under 70 years from major NCD. It is important to monitor the progress that provinces and districts are making with reducing the burden of CVD. The CHI will be used for tracking these actions.

The CHI is useful for policy-makers to evaluate the current cardiovascular health situation and changes to population cardiovascular health through a direct and scientific index system. Using the CHI, policy-makers can form on a decision-making basis ways to reduce early deaths caused by CVD, and to effectively monitor, track and improve the early death situation cause by CVD. Further, the indices can show the effect of government policies and investment in cardiovascular health. Since each province grapples with the local prevalence situation of CVD, the monitoring system differs among provinces, making inter-province comparison difficult, especially whether a province is doing well (or not) in controlling CVD morbidity, the prevention and control of CVD risks or CVD treatment. With the CHI, national and provincial governments can make comparisons with other provinces and which dimension has a relatively lower mark within each province, allowing targeted decision making and improvement. The establishment of the CHI also will become an effective public health instrument, and it is anticipated that the use and generalization of CHI will have influence policy-makers, academic institutions, social organizations, private sectors and international organizations; improve public awareness of CVD prevention and control; help evaluate the implementation of WHO’s policies on chronic diseases and CVD; measure progress towards Healthy China 2030; and promote prevention and control of CVD in China.

The CHI will also address the current issues of uncertain or incorrect data sources CVD at province level, which leads to distortion of results and reduction of credibility. To avoid these situations, we suggest strengthening and improving the monitoring of Provincial-level CVD and data usage, and to adjust the selected indices and weights for Province-level circumstances. In the future, we will calculate and periodically publish the national CHI score and the provincial CHI scores. The next step is to carry out research on the regional and municipal level CHI evaluation system. According to the results of the calculation, the CVD prevention and control measures are formulated in the pilot cities.

### Limitation

One of the limitations of this innovative study is that morbidity rate of atrial fibrillation had not been included in prevalence of CVD dimension. The main risk of atrial fibrillation is thrombosis and embolism, which increases the risk of stroke and death. There is lack of epidemiological monitoring data for atrial fibrillation in China. Considering the availability of indicators, morbidity rate of atrial fibrillation has not been used as an indicator in this study. We hope that we can obtain high-quality atrial fibrillation data and make CHI more comprehensive and scientific in the future. Another limitation is the low response rate of clinicians during the Delphi consultation. For every round of Delphi, we gave experts one month to feedback. The main reasons for the low response rate include the busy work of the clinicians and a larger number of clinicians we invited for the Delphi consultations (around 100 each round). While in the returned Delphi questionnaires, there was a high consistency in judging indicators (scores of all indicators were above 8 points, and the degree of dispersion was low). We also organized core experts’ face-to-face consultation and discussions, to a certain extent, to made up for the low response rate of clinicians.

## Conclusion

China CHI is a comprehensive evaluation system raised to effectively control the prevalence of CVD. A comprehensive score of CHI help us to raise the awareness of CVD in a more intuitive and simple way. There are 52 indicators of 5 dimensions in CHI evaluation system that would be used to evaluate the situation of CVD from prevalence of CVD, exposure of risk factors, prevention and control of risk factors, treatment of CVD and public health policy and service ability. These dimensions and indicators allowed different CVD researchers to focus on specific elements. It is highly recommended to collect data and calculate the scores of CHI over several years to identify the CVD changes and to confirm the effect of related strategies and interventions on it.

## Abbreviations

ACC: American College of Cardiology
AHA: American Heart Association
AHP: Analytical hierarchy process
CHI: Cardiovascular health index
COPD: Chronic obstructive pulmonary disease
CVD: Cardiovascular disease
DALYs: Disability adjusted of life years
HiAP: Health in All Policies
IHD: Ischemic heart disease
PCI: Percutaneous coronary intervention
SD: Standard deviation
STEMI: ST segment Elevation Myocardial Infarction
WHO: World Health Organization
